# National Association of Dental Faculties

**Published:** 1894-10

**Authors:** 


### NATIONAL ASSOCIATION OF DENTAL FACULTIES.

THE Eleventh Annual Session of the National Association of Dental Faculties was held at the Hygeia Hotel, Old Point Comfort, Va., commencing Saturday, August 4, 1894; the President, Dr. H. A. Smith, of Cincinnati, in the chair.

The resignation of Dr. J. E. Cravens as secretary was accepted, and Dr. Louis Ottofy, of Chicago, was made secretary *pro tem.*

The following faculties were represented:

*Baltimore College of Dental Surgery.*—B. Holly Smith.

*Boston Dental College.*—J. A. Follett.

*Chicago College of Dental Surgery.*—T. W. Brophy.
*Harvard University, Dental Department.*—Thomas Fillebrown.
*Kansas City Dental College.*—J. D. Patterson.
*Missouri Dental College.*—A. H. Fuller.
*New York College of Dentistry.*—Frank Abbott.
*Ohio College of Dental Surgery.*—H. A. Smith.
*Pennsylvania College of Dental Surgery.*—C. N. Peirce.
*Philadelphia Dental College.*—S. H. Guilford.
*State University of Iowa, Dental Department.*—W. O. Kulp.
*University of Michigan, Dental Department.*—J. Taft.
*University of Pennsylvania, Dental Department.*—James Truman.
*Vanderbilt University, Dental Department.*—H. W. Morgan.
*Louisville College of Dentistry.*—F. Peabody.
*Southern Medical College, Dental Department.*—C. V. Rosser.
*University of Tennessee, Dental Department.*—J. P. Gray.
*University of Maryland, Dental Department.*—F. J. S. Gorgas.
*Royal College of Dental Surgeons of Ontario.*—J. B. Willmott.
*Columbian University, Dental Department.*—H. C. Thompson.
*Northwestern University Dental School.*—G. H. Cushing.
*American College of Dental Surgery.*—Louis Ottofy.
*National University, Dental Department.*—J. Roland Walton.
*College of Dentistry, University of Minnesota.*—T. E. Weeks.

The following schools were admitted to membership during the meeting:

*Western Reserve University, Dental Department, Cleveland, O.*—H. L. Ambler.

*Western Dental College, Kansas City, Mo.*—D. J. McMillen.

With reference to the application of Howard University, Dental Department, the Executive Committee recommended that in consequence of changes in and inadequacy of its dental department the application be rejected. The report was adopted.

The report of the Executive Committee recommending the admission of the University of Buffalo, Dental Department, to membership, was amended by the addition of the following clause, and then adopted: "When the honorary degrees conferred on Messrs. Southwick and Howard are returned to the University and revoked, and official notification of such revocation filed with the secretary of this Association."[1]

---

[1] The Dental Department of the University of Buffalo complied with these conditions on August 13, 1894, and is therefore admitted into full membership. —Louis Ottofy, *Secretary.*

The amendment to Rule 5 offered by Dr. Hunt last year, making the rule read as follows, was adopted unanimously:

"(5) STANDING OF UNDERGRADUATES IN MEDICINE.—Undergraduates of reputable medical colleges, who have regularly completed one full scholastic year, having attended at least seventy-five per cent. of a five-months' term, and passed a satisfactory examination in the studies of the freshman year, may be admitted to the junior grade in colleges of this Association, subject to other rules governing admission to that grade."

The following new applications for membership, with their recommendations, were reported by the Executive Committee:

Dental Department, Cleveland University of Medicine and Surgery, Cleveland, Ohio. Recommended by Drs. J. Taft and J. E. Garretson.

Cincinnati College of Dental Surgery, Cincinnati, Ohio. Recommended by Drs. James Truman, T. W. Brophy, F. J. S. Gorgas, and J. A. Follett.

Birmingham Dental College, Birmingham, Ala. Recommended by Drs. H. W. Morgan and C. V. Rosser.

University College of Medicine, Dental Department, Richmond, Va. Recommended by Drs. F. J. S. Gorgas and H. W. Morgan.

Atlanta Dental College, Atlanta, Ga. Recommended by Dr. H. W. Morgan and the Faculty of the University of Tennessee, Dental Department.

The amendment to By-law 4, which was offered by Dr. Hunt last year, was laid on the table.

The resolution offered last year by Dr. Sudduth, directing the addition of Latin and physics to the entrance examination, was also laid on the table.

The special committee appointed last year to consider the matter of the vote of censure passed upon the Baltimore College of Dental Surgery reported, through its chairman, Dr. C. N. Peirce, recommending that no further action be taken. The report was adopted.

Dr. Louis Ottofy offered a recommendation, which was adopted, that all colleges, members of this Association, shall increase the college course of 1895–96 to not less than six months.

The following resolution from the Executive Committee was adopted:

*Resolved*, That any college or colleges making application for membership in the National Association of Dental Faculties shall be required to secure and present to the Executive Committee the approval and indorsement of the Board of Dental Examiners of the State (where such boards exist) in which such colleges are located, before their application can be considered.

The following from the Executive Committee was laid on the table:

*Resolved*, That we regard it as inconsistent for any member of a Faculty of any college holding membership in this body to at the same time be a member of any State Board of Dental Examiners.

The report of the *ad interim* committee with reference to charges preferred against the University of Maryland, Dental Department, was referred to the Executive Committee, which reported as follows:

"Your Executive Committee respectfully report that they find that the University of Maryland, Dental Department, in the reception of certain students did violate the regulations of this Association through a misapprehension of the rules, as it is interpreted by your committee that the regular sessions of all colleges close with their commencement exercises."

The report was adopted.

Dr. Guilford moved that Rule 11, page 11 of the "History," be understood to mean that students coming from a college not a member of this Association will not be given credit for any time spent in such institution.

The annual dues were increased from three dollars to five dollars on motion of Dr. Cushing, the increase to take effect in 1895–96.

On motion of Dr. Truman, the special Committee on Preliminary Examinations was instructed to have prepared by a competent person and present at the next annual meeting a list of questions of a standard covering every branch required in the grammar schools up to the point of admission to the high schools.

On motion of Dr. Abbott, it was ordered that each college should each year present its announcement, noting any changes, the secretary to note and publish all important changes in the annual report of the Association.

On motion of Dr. Morgan, all the schools were required to comply with the rule regarding dissections.

On motion of Dr. Ottofy, a committee of three was appointed to revise the constitution and by-laws, with the further instruction, on motion of Dr. Truman, to drop the qualifying term "by."

The following were introduced, and under the rules lie over till next year:

By Dr. Peirce:

*Resolved*, That, in view of the recommendation of the Executive Committee, this Association will require that all colleges, members of this Association, shall extend the term of the session of 1896–97, and of succeeding sessions, to not less than seven months each.

By Dr. Truman :

*Resolved*, That on and after the session of 1898–99 the regular session of each of the colleges belonging to this Association shall be extended to four years.

By Dr. Ottofy :

Beginning with the session of 1896–97, the examinations conducted by the colleges of this Association shall be in the English language only.

By Dr. Ottofy :

Beginning with the session of 1895–96, no college shall be permitted to retain membership in this Association if it is conducted or managed, in whole or in part, by any person or persons who do not practise dentistry in accordance with well-recognized and generally accepted forms, generally known as dental ethics, or if they are owned in whole or in part by men or women who are engaged in disreputable dental practice, or if any college have upon its list of trustees, the Faculty, demonstrators, or in any other capacity, any one who does not practise dentistry in accordance with the principles above mentioned. This shall refer to dentists only.

By Dr. Ottofy :

Beginning with the session of 1896–97, the following shall be the requirements for the admission of students to the colleges of this Association :

*a.* A certificate of having successfully completed at least one full year's course of study in the collegiate department of any college or university registered by the regents of the State of New York as maintaining a satisfactory standard.

*b.* A certificate of having passed, in a registered institution, examinations equivalent to the full collegiate course of the freshman year, or to a completed three years' academic course.

*c.* Regents of the State of New York pass cards for any forty-eight counts.

*d.* A certificate of having passed the matriculation examinations of any university in Great Britain or Ireland, or of having completed a course of study recognized as an equivalent therefor.

*e.* A certificate of graduation from any registered gymnasium in Germany, Austria, or Russia.

*f.* A certificate of the successful completion of a course of five years in a registered Italian *ginnasio* and three years in a *liceo.*

*g.* The bachelor's degree in arts or science, or substantial equivalents, from any registered institution in France or Spain.

*h.* Any credential from a registered institution, or from the government in any foreign state or country, which represents the completion of a course of studies equivalent to graduation from a registered New York high school, academy, or from a registered Prussian gymnasium.

By Dr. Gray :

*Resolved*, That law 7 of the by-laws, which now reads, "attendance upon three full courses of not less than five months each in separate years shall be required before examination for graduation," be amended by substituting "six" instead of "five," to take effect on and after the year 1896–97.

By Dr. Willmott:

*Resolved,* That at least twenty-nine months intervene between the beginning of the freshman year and the date of graduation.

The Committee on Text-Books presented the following report, which was adopted:

Your Committee on Text-Books would report that only two works of this character have been presented for its consideration:

One, a work on "Dental Anatomy and Pathology," by Dr. C. F. W. Bödecker, of New York, seven hundred pages; and the other, a smaller and less pretentious work of about seventy-five pages, on "Operative Techniques," by Dr. Thomas E. Weeks, of Minneapolis.

Both of these works are in press and nearly completed. The treatment of their subjects is full, clear, and concise, and the illustrations numerous, well executed, and for the most part entirely new.

Your committee would therefore recommend these two works for indorsement as text-books.

Suitable resolutions regarding the death of Drs. R. B. Winder and W. H. Eames were presented and adopted, and the secretary was instructed to communicate a copy to their respective families.

The following officers were elected for the ensuing year: Frank Abbott, President; S. H. Guilford, Vice-President; Louis Ottofy, Secretary; H. W. Morgan, Treasurer; J. Taft, B. Holly Smith, and Thomas Fillebrown, Executive Committee; James Truman, Truman W. Brophy, and Francis Peabody, *Ad Interim* Committee.

Dr. Abbott, the newly-elected president, was installed, and appointed the following committees:

*Committee on Schools.*—J. A. Follett, F. J. S. Gorgas, Louis Ottofy, C. N. Peirce, and Truman W. Brophy.

*Committee on Text-Books.*—J. D. Patterson, A. O. Hunt, J. B. Willmott, T. E. Weeks, and J. P. Gray.

